# HMGB2 Impacts Cisplatin-Induced DNA Adduct Processing in Chemoresistant Ovarian Cancer Cells

**DOI:** 10.3390/genes17080916

**Published:** 2026-08-02

**Authors:** Van Huynh, Guliang Wang, Karen M. Vasquez

**Affiliations:** Division of Pharmacology and Toxicology, College of Pharmacy, The University of Texas at Austin, Dell Pediatric Research Institute, 1400 Barbara Jordan Boulevard, Austin, TX 78723, USA; vh5427@my.utexas.edu (V.H.); guliang.wang@austin.utexas.edu (G.W.)

**Keywords:** HMGB2, cisplatin, ovarian cancer, DNA damage response, DNA repair, interstrand crosslinks, chemoresistance

## Abstract

**Background/Objectives:** Cisplatin is used in the treatment of ovarian cancer; however, the development of resistance, often due to the efficient repair of cisplatin-induced DNA damage, remains a major barrier to effective therapy. Among these lesions, DNA interstrand crosslinks (ICLs) are particularly cytotoxic because they prevent DNA replication and transcription. HMGB2, a member of the high-mobility group box (HMGB) protein family, can bind DNA lesions and has been implicated in genome maintenance and DNA repair. This study investigated whether HMGB2 contributes to the processing of cisplatin-induced DNA damage and modulates cisplatin sensitivity in human ovarian cancer cells. **Methods:** HMGB2 expression was suppressed by siRNA in cisplatin-sensitive A2780 and cisplatin-resistant CP70 human ovarian cancer cells. Cellular responses to cisplatin were assessed using clonogenic survival assays, cell cycle analysis, Western blotting, slot blot analysis, and modified alkaline comet assays. **Results:** HMGB2 depletion reduced clonogenic survival in cisplatin-resistant CP70 cells and increased the sub-G1 population, indicating enhanced apoptotic DNA fragmentation following cisplatin treatment in both cell lines. Depletion of HMGB2 resulted in increased persistence of cisplatin–DNA adducts and impaired ICL processing, as demonstrated by persistent DNA damage over time and reduced ICL unhooking efficiency. DNA damage response signaling following cisplatin treatment was also altered by HMGB2 depletion in both cell lines, whereas the expression levels of key DNA repair proteins were unchanged. **Conclusions:** Our findings demonstrate that HMGB2 is involved in the cellular response to cisplatin treatment by promoting the efficient processing of cisplatin DNA adducts, particularly ICLs, thereby modulating cisplatin sensitivity in human ovarian cancer cells. These findings suggest that targeting HMGB2 may serve as a potential therapeutic strategy for overcoming cisplatin resistance in ovarian cancer.

## 1. Introduction

Ovarian cancer remains challenging to treat and can result in poor prognosis due to late-stage diagnosis and high recurrence rates [1]. In 2026, over 21,000 new ovarian cancer cases are predicted, with over 12,000 deaths expected in the United States [1]. Platinum-based chemotherapy, particularly cisplatin, remains a first-line treatment for ovarian cancer [2]. Although many patients initially respond well to platinum therapy, the majority eventually relapse and develop chemoresistance, which is the major cause of treatment failure and poor overall outcome [3,4].

Cisplatin toxicity is primarily mediated by the formation of DNA lesions, including intrastrand and interstrand cross-links (ICLs) [5], which can interfere with DNA replication and transcription and activate DNA damage response (DDR) signaling pathways [6]. ICLs are particularly cytotoxic because they covalently link both DNA strands and require complex mechanisms involving multiple repair proteins for their removal [7]. Upregulation of DDR and repair of cisplatin-induced DNA lesions are major contributors to acquired chemoresistance and are therefore critical for determining cellular sensitivity to cisplatin [8].

The high-mobility group box (HMGB) proteins are non-histone DNA-binding proteins involved in a variety of cellular processes including gene transcription and DNA repair [9,10,11]. HMGB proteins have been shown to bind distorted DNA structures, including cisplatin-modified DNA, suggesting potential roles in cellular responses to cisplatin treatment [10]. Our previous studies demonstrated that the depletion of HMGB3 resensitized cisplatin-resistant ovarian cancer cells to cisplatin treatment by reducing ATR/CHK1 expression and increasing apoptosis, while the depletion of HMGB1 enhanced cisplatin sensitivity by altering DDR signaling and reducing the efficiency of cisplatin–DNA adduct processing [12,13]. These findings indicate that members of the HMGB family contribute to cellular responses to cisplatin through several mechanisms.

HMGB2, a member of the HMGB family, shares structural similarity with HMGB1 and HMGB3 [14] and plays roles in DNA transcription, replication, repair, cell-cycle progression, and proliferation [11,15,16,17,18]. In several cancer types, HMGB2 overexpression has been associated with poor prognosis, tumor progression, and resistance to DNA-damaging therapies [19,20,21]. Studies in hepatocellular carcinoma and glioma suggest that HMGB2 promotes survival following genotoxic stress and may participate in DNA repair-related processes [19,20]. However, whether HMGB2 is involved in cisplatin-induced DNA damage processing and cisplatin resistance in ovarian cancer remains unclear.

Here, we investigated the role of HMGB2 in the responses to cisplatin treatment using cisplatin-sensitive (A2780) or cisplatin-resistant (A2780/CP70) human ovarian cancer cells. We examined the effects of HMGB2 depletion on clonogenic survival, apoptosis, DDR signaling, cisplatin–DNA adduct removal, and, in particular, ICL processing following cisplatin treatment. Our results demonstrate that HMGB2 modulates the efficient processing of cisplatin-induced DNA lesions and influences cisplatin sensitivity in ovarian cancer cells. These results indicate that HMGB2 can modulate DDR to platinum-based chemotherapy and provide new insight into mechanisms underlying chemoresistance in ovarian cancer.

## 2. Materials and Methods

### 2.1. Cell Culture and Maintenance

Human ovarian cancer cell lines A2780 (cisplatin-sensitive; RRID: CVCL_0134) and A2780/CP70 (CP70, cisplatin-resistant; RRID: CVCL_0135) were utilized as previously described [13]. Briefly, the cell lines were purchased from the American Type Culture Collection (ATCC, Manassas, VA, USA) and were confirmed via short tandem repeat (STR) profiling. Cells were grown in RPMI-1640 medium supplemented with 10% fetal bovine serum at 37 °C in a humidified incubator with 5% CO_2_. Cells were passaged at ~80–90% confluence using 0.25% trypsin-EDTA. A2780 cells were subcultured at ratios between 1:3 and 1:5, while CP70 cells were maintained between 1:3 and 1:4. An amount of 1 µM of cisplatin was added to the growth media every third passage to preserve the resistant phenotype of the CP70 cells. For the current study, experiments were conducted once cultures reached 70–80% confluence in the third passage.

### 2.2. Cisplatin Cytotoxicity and IC50 Measurement

Cisplatin (1 mg/mL in 140 mM NaCl) was freshly prepared prior to each experiment, as previously described [13]. To determine the half-maximal inhibitory concentration (IC50) of cisplatin in ovarian cancer cell lines, A2780 and CP70 cells were plated at 1.5 × 10^4^ cells per well in 96-well plates. Following a 24-h attachment period, cells were exposed to varying concentrations of cisplatin (half-serial dilutions from 100 μM) for 72 h. Cell viability was quantified using a colorimetric MTT assay (CellTiter 96™ kit, Promega, Madison, WI, USA) following the manufacturer’s protocol. Briefly, post-treatment cells were washed with PBS and incubated with MTT reagent for 3 h at 37 °C. Dyes were subsequently dissolved overnight using the provided solubilization buffer. Absorbance was measured at 570 nm (Synergy H1; BioTek Instruments, Winooski, VT, USA), and the IC50 was calculated from the resulting dose–response curves. For experimental treatments, the cells were exposed to cisplatin at concentrations optimized for each assay. Following drug exposure, cells were either collected immediately or allowed to recover in drug-free medium over a time course to assess DNA damage formation and repair.

### 2.3. siRNA-Mediated HMGB2 Depletion

Depletion of HMGB2 was performed by using an siGENOME SMARTpool siRNA (Dharmacon, Lafayette, CO, USA, Cat. No. M-018981-01-0020), consisting of four individual siRNA sequences targeting the human *HMGB2* transcript. A siGENOME non-targeting siRNA pool (siRNA #2, Dharmacon, Lafayette, CO, USA, Cat. No. D-001210-02-20) was used as the negative control. The A2780 and CP70 cells were transfected using Lipofectamine RNAiMAX (Invitrogen, Carlsbad, CA, USA) according to the manufacturer’s instructions and previously established protocol [22]. Briefly, Lipofectamine RNAiMAX and siRNA were diluted separately in Opti-MEM Reduced Serum Medium (Thermo Fisher Scientific, Waltham, MA, USA) and then combined to allow complex formation for approximately 5–10 min at room temperature. For reverse transfection, the prepared cell suspension in antibiotic-free culture medium was added directly to the siRNA mixture and plated in 60 mm culture dishes. For A2780 cells, transfection was performed using a final siRNA concentration of 40 nM with 8 µL RNAiMAX, whereas CP70 cells were treated with 30 nM siRNA and 7 µL RNAiMAX. After 24 h, the medium was replaced with fresh complete medium for another 24 h. A second forward transfection was then performed to enhance knockdown efficiency. Fresh siRNA in RNAiMAX was prepared as described above and added to cells after the culture medium was removed. Following 24 h of exposure to the transfection mixture, the medium was replaced, and cells were cultured for an additional 24 h before collection for protein analysis or subsequent functional assays. HMGB2 knockdown efficiency was confirmed by Western blot analysis prior to use in subsequent experiments.

### 2.4. Western Blot Analysis

Protein levels were estimated using Western blot analysis using total cell lysates collected at the indicated experimental time points. Cells were isolated by scraping and centrifuging at 13,000 rpm (~17,900× *g*) for 10 min at 4 °C, and the resulting pellets were stored at −80 °C until further processing. Proteins were extracted using cell pellets resuspended in ice-cold RIPA buffer containing protease inhibitor cocktail (cOmplete™, Roche, Basel, Switzerland) and phosphatase inhibitor cocktail (PhosSTOP™, Roche, Basel, Switzerland). Samples were incubated on ice for 1 h and sonicated to ensure complete lysis. Total protein concentration was measured using the Pierce™ BCA Protein Assay Kit (Thermo Fisher Scientific, Waltham, MA, USA). Equal amounts of protein (45 μg) were prepared in 2X Laemmli sample buffer (Bio-Rad, Hercules, CA, USA) and heated at 95 °C for 10 min before loading. Proteins were separated on 4–15% Mini-PROTEAN^®^ TGX™ precast gels (Bio-Rad, Hercules, CA, USA) in Tris-Glycine-SDS running buffer and subsequently transferred onto 0.2 μm nitrocellulose membranes using the Trans-Blot Turbo Transfer System (Bio-Rad, Hercules, CA, USA). Following transfer, membranes were washed and blocked with 3.7% non-fat dry milk prepared in 1X phosphate-buffered saline containing 0.1% Tween-20 (PBST) for 1 h at room temperature and then incubated overnight at 4 °C with primary antibodies against phospho-ATM, phospho-ATR, phospho-CHK1, phospho-CHK2, ATM, ATR, CHK1, CHK2, HMGB2, and β-actin as the loading control (Supplementary Table S1). After washing, membranes were incubated with HRP-conjugated secondary antibodies for 1 h at room temperature. Protein bands were visualized using Clarity Western ECL Substrate (Bio-Rad) and detected with a ChemiDoc XRS+ imaging system (Bio-Rad, Hercules, CA, USA). Densitometric quantification of band intensity was performed using Fiji/ImageJ (National Institutes of Health (NIH), Bethesda, MD, USA; version 2.14.0/1.54f; RRID: SCR_003070).

### 2.5. Colony Formation Assay

A2780, CP70, and siRNA-transfected cells, including HMGB2 siRNA (siHMGB2) and non-targeting siRNA (siNT), were treated with cisplatin at a concentration corresponding to one-fourth of the IC50 previously established for CP70 cells. This concentration remained highly effective against A2780 cells while allowing evaluation of whether HMGB2 depletion increased the sensitivity of the resistant CP70 cells under identical treatment conditions. Untreated cells were included as controls. Following 72 h of treatment, cells were collected and counted using an automated cell counter (Invitrogen, Carlsbad, CA, USA, Cat. No. A49866). A total of 1000 viable cells were then replated into 60 mm culture dishes containing 6 mL of complete growth medium and maintained at 37 °C in a humidified incubator with 5% CO_2_ for 10–14 days to allow colony formation. At the end of the incubation period, colonies were washed with PBS, fixed with 95% ethanol for 10 min, air dried, and stained with 0.125% crystal violet for 30 min at room temperature. Colonies containing 50 or more cells were counted manually. Data were expressed as the total number of colonies formed and presented as mean ± SEM from at least three independent experiments. Statistical analysis was performed using the ordinary two-way ANOVA followed by Sidak’s multiple comparisons test, with significance defined as *p* < 0.05.

### 2.6. Cell Cycle Analysis for Sub-G1 Population Assessment

Cell cycle distribution and apoptotic sub-G1 populations were evaluated by flow cytometry using our previous protocol [13]. Cells were transfected with *HMGB2*-targeting siRNA (siHMGB2) or non-targeting control siRNA (siNT) as described above. Non-treated cells were included as controls. After 72 h of cisplatin exposure, the cells were trypsinized, harvested, counted, and washed twice with ice-cold PBS. Cell pellets were resuspended in cold PBS and fixed with chilled 70% ethanol. Fixed samples were stored overnight at −20 °C prior to analysis. The cells were then collected by centrifugation, washed with cold PBS, and resuspended in propidium iodide (PI) staining solution containing 20 μg/mL PI, 20 μg/mL RNase A, and 0.5% Triton X-100 in PBS. Samples were filtered through a 35-μm nylon mesh to obtain single-cell suspensions and then incubated at 37 °C for 1 h before analysis. DNA content and cell cycle profiles were analyzed using the BD Accuri C6 flow cytometer (BD Biosciences, San Jose, CA, USA, RRID: SCR_019594). The percentage of cells in the sub-G1 phase was quantified using FlowJo software version 10.5.3 (BD Life Sciences, Ashland, OR, USA; RRID: SCR_008520) and used as an indicator of apoptotic DNA fragmentation. Data represented the results from at least three independent biological experiments.

### 2.7. DNA Damage and Repair Analysis via Slot Blot Assay

Cisplatin–DNA adduct formation and removal were evaluated using a slot blot assay based on our previously established protocol [12] with modifications for HMGB2 depletion. Briefly, control and HMGB2-depleted cells were treated with cisplatin for 24 h. Cells were collected immediately after treatment (T0) and after 24 h (T24) or 96 h (T96) of recovery in drug-free medium to assess DNA adduct persistence and removal efficiency. Untreated cells were included as negative controls. Genomic DNA was extracted using the DNeasy Blood & Tissue Kit (Qiagen, Hilden, Germany), and concentrations were verified via NanoDrop spectrophotometer (Thermo Fisher Scientific, Waltham, MA, USA). 600 ng of DNA from each sample was denatured at 100 °C for 10 min, immediately stabilized with cold 2 M ammonium acetate, and immobilized onto a cellulose membrane using a Bio-Dot SF microfiltration system (Bio-Rad, Hercules, CA, USA). Following fixation at 80 °C for 2 h, membranes were blocked and probed overnight at 4 °C with an anti-cisplatin-modified DNA primary antibody (1:15,000; Abcam, Cambridge, UK, CAT. No. ab103261, RRID: AB_10715243). Immuno-complexes were detected using an HRP-conjugated anti-rat secondary antibody (1:5,000, Cell Signaling Technology, Danvers, MA, USA, CAT. No. 7077, RRID:AB_10694715) and visualized with Clarity Western ECL substrate (Bio-Rad, Hercules, CA, USA, CAT. No. 1705061). For a DNA loading control, membranes were subsequently stained with SYBR Gold (1:10,000). Signal intensities were quantified using Fiji/ImageJ (NIH, version 2.14.0/1.54f). Statistical analysis was performed using two-way ANOVA followed by Sidak’s multiple comparisons test in GraphPad Prism version 9 (GraphPad Software, RRID: SCR_002798). Data are presented as mean ± SEM from at least three independent biological replicates.

### 2.8. Analysis of ICL Repair via Alkaline Comet Assay

The alkaline comet assay was performed as previously described in our HMGB1 study [12], with modifications for the present study. Briefly, wild-type, non-targeting siRNA-treated, or HMGB2-depleted cells were treated with 100 µM cisplatin for 1 h, followed by recovery in drug-free medium. Cells were harvested at T10, T24, T48, and T72 (representing hours post-treatment) to monitor the processing of cisplatin-induced ICLs. At each time point, cells were collected, counted, and frozen at a density of 1 × 10^6^ cells/mL in medium containing 10% DMSO and 10% FBS at −80 °C until analysis. The alkaline comet procedure followed an established protocol described by Swift et al. [23]. Briefly, cells (2 × 10^5^) were suspended in 0.8% low-melting-point agarose and cast onto slides pre-coated with 1% agarose. Two gels were prepared per sample; one was treated with 20 μM hydrogen peroxide for 15 min to generate DNA strand breaks, while the paired gel remained untreated. Following solidification and H_2_O_2_ treatment, slides were incubated overnight in a chilled lysis buffer (2.5 M NaCl, 0.1 M Na_2_EDTA, 10 mM Tris, 1% Triton X-100, 10% DMSO; pH 10). DNA was allowed to unwind in an alkaline electrophoresis buffer (0.3 M NaOH, 1 mM Na_2_EDTA; pH 12.5) for 30 min, followed by electrophoresis at 1 V/cm for 25 min at 4 °C. After neutralization and ethanol dehydration, slides were dried overnight at room temperature. After rehydration, DNA was stained with SYBR Gold (1:10,000) and imaged using a Zeiss AxioObserver Z1 microscope (Carl Zeiss Microscopy, Oberkochen, Germany). At least 100 comets per sample were analyzed using CometScore 2.0 (Red Hoover Solutions, Salem, NH, USA). The Olive tail moment was used to assess DNA migration. Cisplatin-induced ICLs were quantified based on the decrease in tail moment (DTM) in H_2_O_2_-treated samples. The T10 sample represents the highest ICL level. The relative ICL unhooking was determined by comparing the %DTM at recovery time points (T24–T72) to the peak ICL levels observed at T10, as previously described [12]. At least three independent biological replicates were performed for statistically significant analysis.

### 2.9. Statistical Analysis

Statistical analyses and graphics were performed using GraphPad Prism software version 9 (GraphPad Software, San Diego, CA, USA). The data are presented as mean ± SEM (standard error of the mean) from at least three independent biological replicates unless otherwise indicated. Statistical significance between groups was determined using Student’s *t*-test or ordinary two-way ANOVA followed by Sidak’s multiple comparisons test, depending on the experimental design. Differences were considered statistically significant when *p* < 0.05. Specific statistical details are provided in the figure legends.

## 3. Results

### 3.1. HMGB2 Depletion Sensitizes Cisplatin-Resistant Ovarian Cancer Cells to Cisplatin Treatment

**Figure 1:**
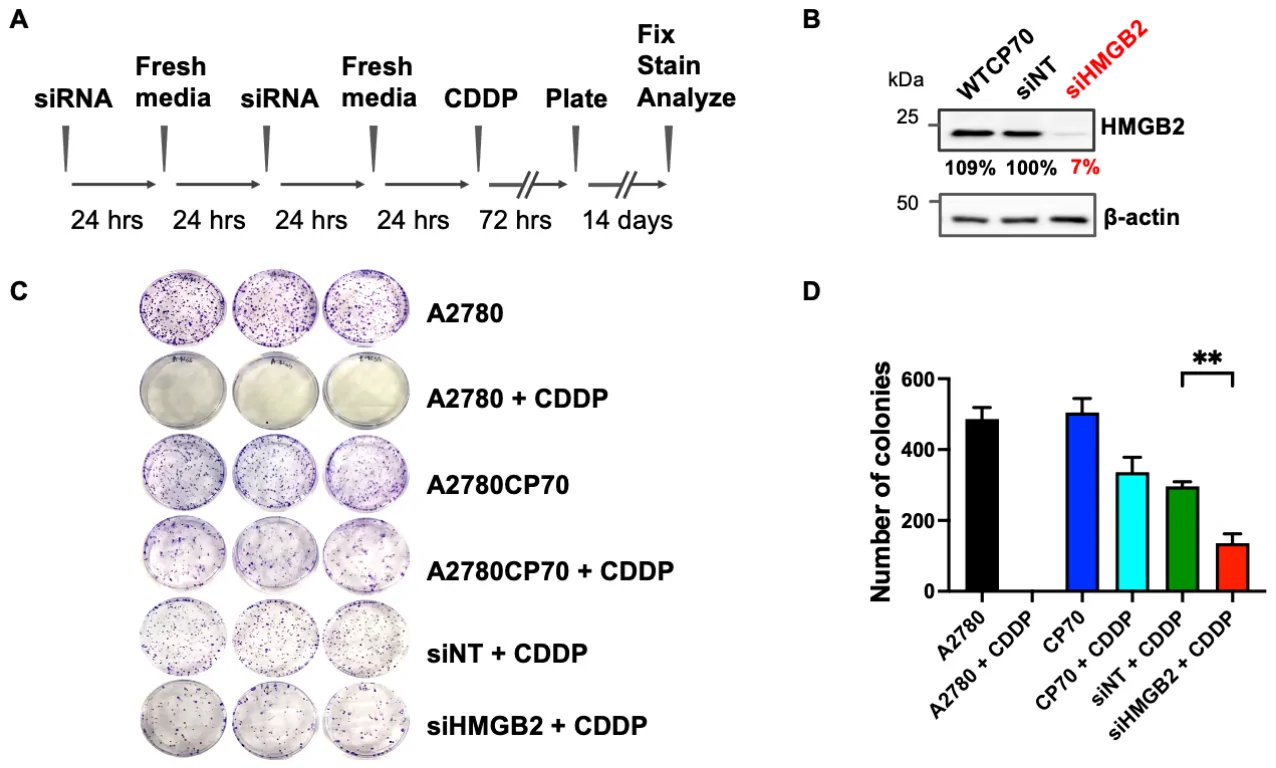
Effects of HMGB2 depletion on cisplatin sensitivity in chemoresistant human ovarian cancer cells. (**A**), Workflow for clonogenic survival assays. Cisplatin-resistant A2780CP70 (CP70) cells were transfected with siRNA targeting *HMGB2* (siHMGB2) or a non-targeting control siRNA (siNT) before exposure to 1.5 µM cisplatin (CDDP) for 72 h. After treatment, ~1000 cells were replated in cisplatin-free medium and incubated at 37 °C with 5% CO_2_ for 14 days to assess colony formation. (**B**), Immunoblot analysis validated the efficiency of HMGB2 depletion, with β-actin used as a loading control. (**C**), Representative images of clonogenic growth from each treatment condition. A2780, cisplatin-sensitive human ovarian cancer cells. (**D**), Quantification of colony formation. HMGB2 inhibition significantly reduced clonogenic survival in cisplatin-treated CP70 cells compared to the control. Data represent mean ± SEM from at least three independent experiments (** *p* < 0.01, n = 3).

To determine if HMGB2 is involved in cisplatin resistance, we established an siRNA-mediated knockdown protocol using cisplatin-sensitive A2780 and cisplatin-resistant CP70 human ovarian cancer cell lines, followed by clonogenic survival analysis as outlined in Figure 1A. CP70 cells were transfected with *HMGB2*-targeting siRNA (siHMGB2) or non-targeting control siRNA (siNT), followed by cisplatin treatment for 72 h. Cisplatin was used at a concentration approximately four times lower than the IC50 value established for CP70 cells via MTT assays. Cells were then replated and allowed to form colonies over 10–14 days. Knockdown efficiency was confirmed by Western blot analysis, which showed more than 90% reduction in HMGB2 protein levels (Figure 1B). The results showed that cisplatin treatment completely inhibited colony formation in A2780 cells. In contrast, cisplatin-resistant CP70 cells and siNT-transfected controls maintained colony growth with only minor reduction after treatment. Importantly, colony survival was reduced to 27% in the siHMGB2 group compared with 59% in the siNT control group (*p* < 0.01) (Figure 1C,D). These results indicate that HMGB2 supports clonogenic survival following cisplatin treatment and that its depletion restores cisplatin sensitivity in resistant ovarian cancer cells.

### 3.2. Cell Cycle Analysis Reveals Increased Sub-G1 Populations in Cisplatin-Treated HMGB2-Depleted Cells

**Figure 2:**
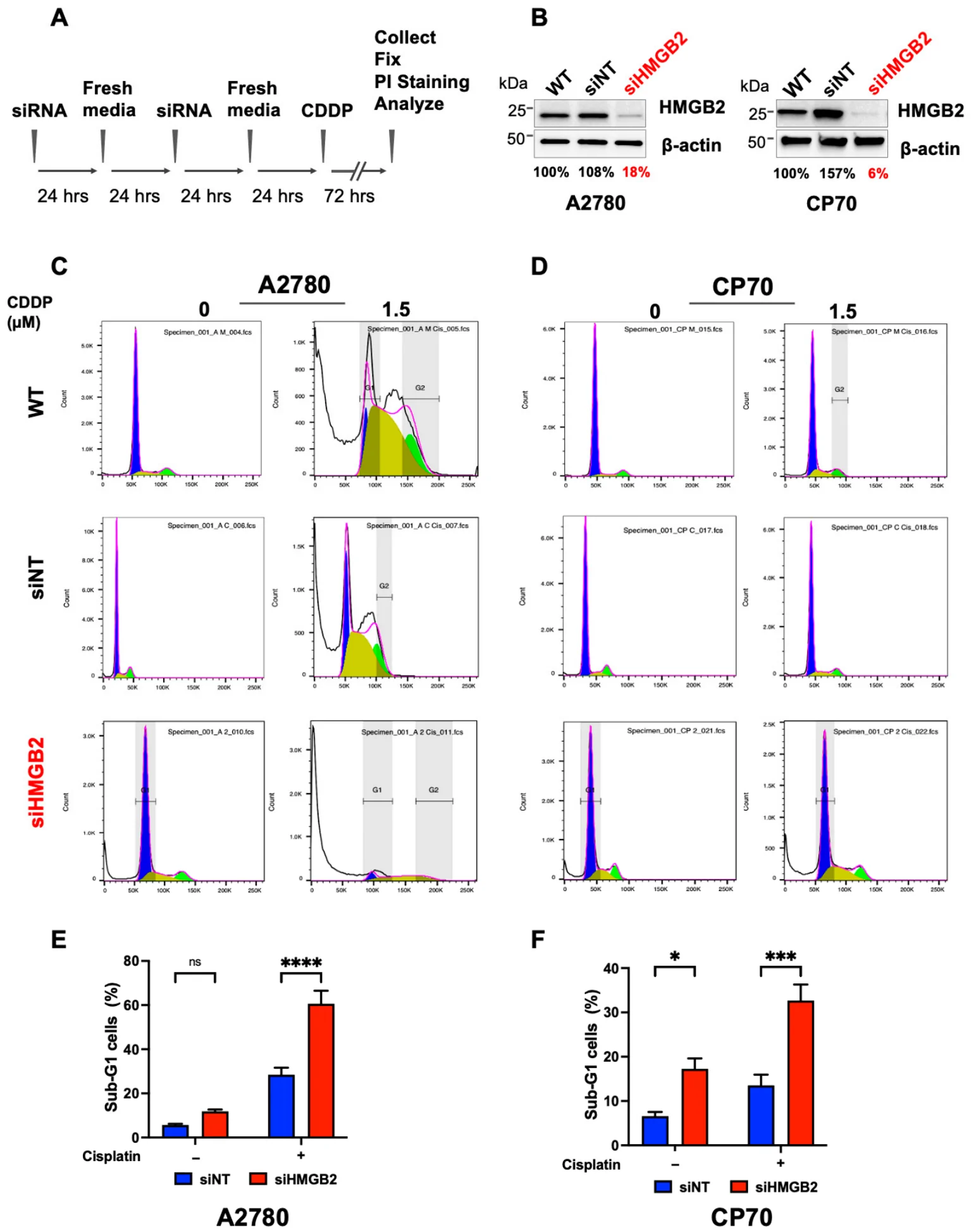
Effects of HMGB2 depletion on cell cycle distribution and the sub-G1 population following cisplatin treatment. (**A**), Overview of the experimental design for cell cycle analysis. A2780 and CP70 cells were treated with siHMGB2 or siNT, followed by incubation with 1.5 µM cisplatin in the media for 72 h. The cells were then fixed with 75% ethanol, stained with propidium iodide (PI), and subjected to flow cytometry analysis. (**B**), HMGB2 knockdown efficiency was validated by Western blot; β-actin was included as a loading control. (**C**,**D**), Representative cell cycle distribution obtained from FlowJo analysis for A2780 and CP70, respectively. The sub-G1 fraction was used as an indicator of apoptotic DNA fragmentation. (**E**,**F**), Quantification of the sub-G1 fraction. The data were analyzed using FlowJo software and are indicated as the mean ± SEM from three independent experiments. Statistical significance was obtained using ordinary two-way ANOVA with Sidak’s multiple comparisons test (* *p* < 0.05; *** *p* < 0.001; **** *p* < 0.0001; ns, not significant; n = 3).

To evaluate the effect of HMGB2 depletion on cisplatin-induced apoptosis, HMGB2-depleted (siHMGB2) and control (siNT) A2780 and CP70 cells were treated with cisplatin for 72 h at a concentration approximately fourfold lower than the IC50 value of the CP70 cells. The sub-G1 fraction was evaluated via flow cytometry using propidium iodide (PI) staining following treatment as outlined in Figure 2A. HMGB2 knockdown efficiency was determined by immunoblotting in both cell lines (Figure 2B). In A2780 cells, cisplatin treatment resulted in ~28% of sub-G1 cells in the siNT group. HMGB2 depletion significantly increased this fraction to 60%, consistent with increased apoptotic DNA fragmentation (Figure 2C,E). In CP70 cells, HMGB2 depletion alone significantly increased the sub-G1 population (17%) compared with the untreated siNT control (7%) (*p* < 0.05). Notably, cisplatin treatment alone did not significantly increase the sub-G1 population in siNT cells. In contrast, cisplatin treatment significantly increased the sub-G1 population in HMGB2-depleted cells from 17% to 33% (*p* < 0.001), resulting in an approximately 2.5-fold higher sub-G1 fraction than that observed in cisplatin-treated siNT cells (Figure 2D,F). These results suggest that HMGB2 depletion increases the sub-G1 population following cisplatin treatment in both A2780 and CP70 ovarian cancer cells, consistent with enhanced apoptotic DNA fragmentation.

### 3.3. Effects of HMGB2 Depletion on DDR Signaling and DNA Damage Repair After Cisplatin Treatment

**Figure 3:**
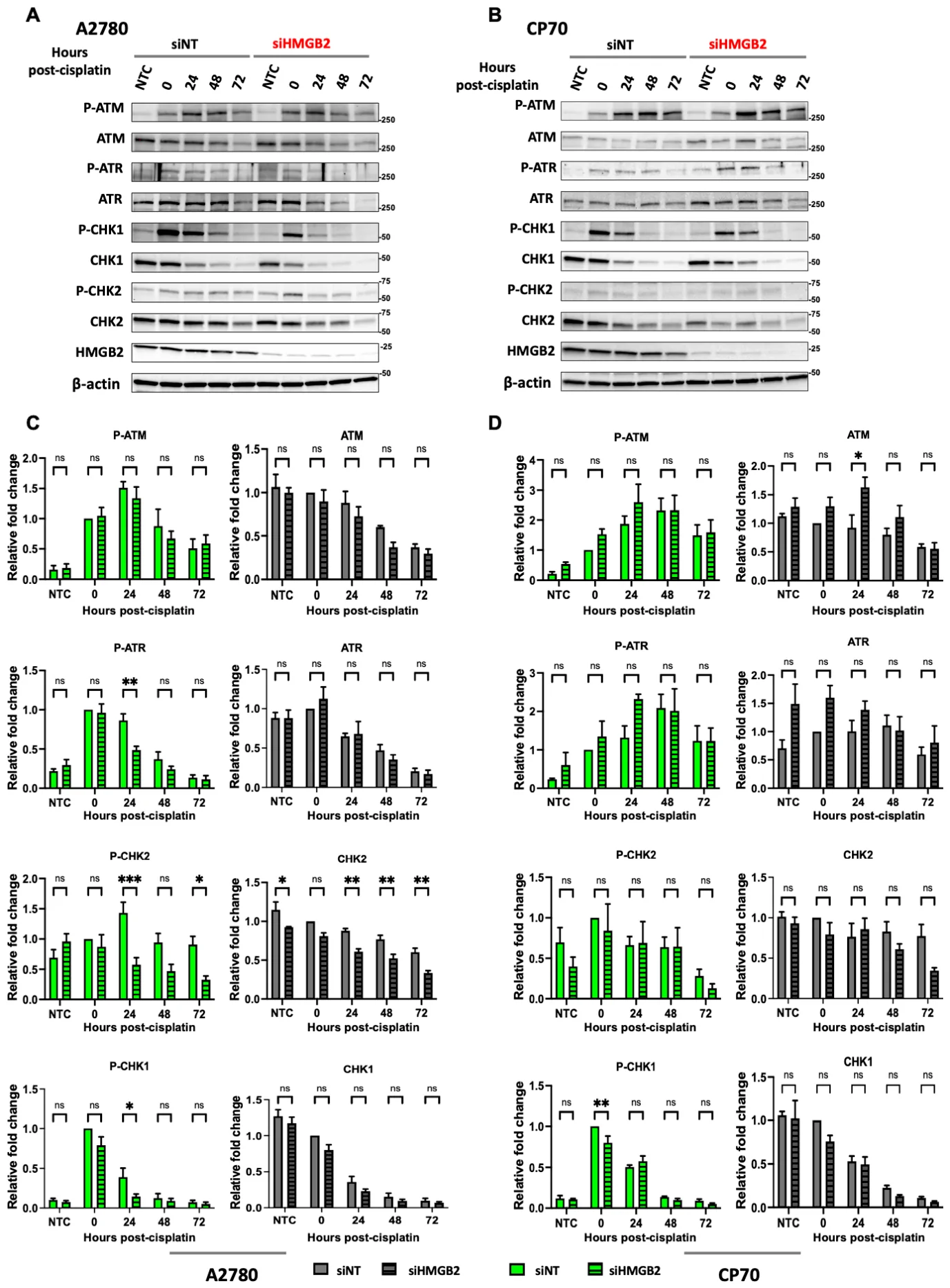
Modulation of DNA damage response and DNA repair proteins following cisplatin treatment by HMGB2 depletion in human ovarian cancer cells. (**A**,**B**), Representative immunoblot panels showing the expression levels of DNA damage response (DDR) proteins from the ATM/CHK2 and ATR/CHK1 pathways in A2780 and CP70 cells. Cells were transfected with siRNA before exposure to cisplatin-containing medium at the IC50 concentration for 24 h. After treatment, the cisplatin-containing medium was replaced with fresh medium, and cells were collected immediately for the 0-h time point or after 24, 48, and 72 h of recovery for Western blot analysis. Both phosphorylated and total protein levels were examined to assess changes in DDR signaling. (**C**,**D**), Quantification of protein band intensities following cisplatin treatment in siHMGB2- and siNT-treated A2780 and CP70 cells, respectively. Protein levels were normalized to β-actin and then to the value of the 0-h time point in the siNT control to determine relative fold changes. Data are presented as mean ± SEM from at least three independent experiments. Statistical significance was determined by two-way ANOVA with Sidak’s multiple comparisons test (* *p* < 0.05; ** *p* < 0.01; *** *p* < 0.001; ns, not significant).

Alterations in DDR signaling and DNA repair activity represent major mechanisms underlying cisplatin resistance [24,25,26]. Thus, we investigated the impact of HMGB2 depletion on these processes. siNT- and siHMGB2-treated A2780 and CP70 cells were treated with cisplatin at the IC50 concentration for 24 h, and recovered in drug-free medium. Cells were collected at 0, 24, 48, and 72 h to assess DDR and DNA damage repair protein levels by immunoblotting (Figure 3A,B). Densitometric analysis using Fiji software version 2.14.0/1.54f is presented in Figure 3C,D, and Supplementary Figure S1C,D for A2780 and CP70 cells, respectively. The protein levels were normalized to β-actin, and relative changes were calculated based on the 0 h time point in the siNT group. Compared with the siNT-treated A2780 cells, p-ATM, total ATM, ATR, and CHK1 levels were not significantly altered in HMGB2-depleted A2780 cells, whereas p-ATR and p-CHK1 were significantly reduced at 24 h (to 5% and 37% of control, respectively). In addition, p-CHK2 levels were decreased at 24 (45%) and 72 (36%) h, and total CHK2 levels were consistently reduced across multiple time points (Figure 3C). These changes indicate that HMGB2 depletion affects selected checkpoint signaling components without altering upstream DDR protein levels. In contrast, CP70 cells exhibited minimal changes in DDR signaling following HMGB2 depletion. Most DDR proteins tested, including p-ATR, p-CHK2, and CHK2, which were reduced in HMGB2-depleted A2780 cells were not significantly different from siNT controls across all time points. An increase in total ATM (~1.7 fold, *p* < 0.01) was observed at 24 h, and p-CHK1 levels were reduced at the 0 h time point; however, no consistent changes were observed in the phosphorylation or levels of the DDR proteins examined following HMGB2 depletion (Figure 3D).

To further evaluate whether HMGB2 depletion affects DNA damage accumulation and the amount of DNA repair-associated proteins, the levels of γH2AX and selected DNA repair proteins involved in cisplatin–DNA lesion processing were analyzed (Supplementary Figure S1). In A2780 cells, γH2AX levels increased over time following cisplatin treatment, but no significant differences were observed between siNT- and siHMGB2-treated groups. Similarly, the levels of XPF, ERCC1, and NBS1 were not significantly altered across all time points. In CP70 cells, γH2AX and ERCC1 levels were comparable between the siNT and siHMGB2 groups following cisplatin treatment. However, an increase in XPF at 24 h (~1.2 fold) and reductions in NBS1 at 0 h and 48 h were observed in HMGB2-depleted cells. No significant changes in γH2AX were detected under these conditions. However, because γH2AX reflects multiple forms of DNA damage and cellular stress signaling, this result does not exclude differences in cisplatin-induced DNA lesion persistence or processing.

These findings indicate that HMGB2-depletion in cisplatin-treated A2780 cells altered the phosphorylation of ATR, CHK1, and CHK2. In contrast, the effects of HMGB2 depletion on the cisplatin-induced DDR in cisplatin-resistant CP70 cells were less pronounced, with only a transient reduction in p-CHK1 levels immediately after treatment and an increase in total ATM levels observed. Overall, these changes do not indicate a dramatic global disruption of DDR signaling. Importantly, these relatively limited changes in the phosphorylation and levels of DDR components contrast with the significant increase in cisplatin sensitivity observed upon HMGB2 depletion, suggesting that HMGB2 may influence the cellular response to cisplatin through modulating the efficiency and/or coordination of DNA lesion processing following cisplatin treatment.

### 3.4. HMGB2 Depletion in Human Ovarian Cancer Cells Results in the Accumulation and Persistence of Cisplatin–DNA Adducts

**Figure 4:**
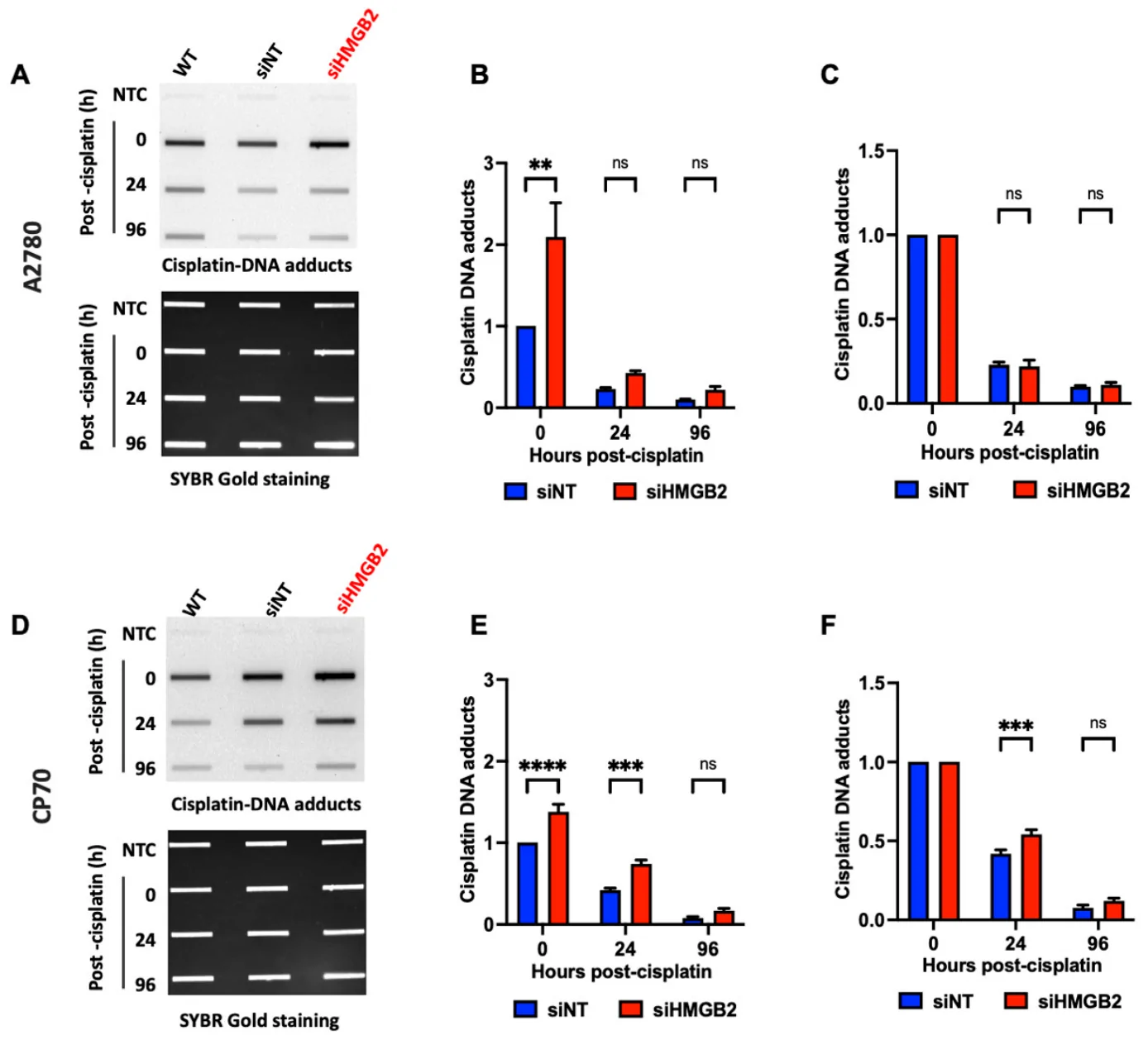
HMGB2 depletion impacts cisplatin-induced DNA adduct processing in human ovarian cancer cells. (**A**,**D**), Detection of cisplatin–DNA adducts by slot blot analysis in A2780 and CP70 cells, respectively. Cells were transfected with siHMGB2 or siNT and treated with cisplatin at half of the IC50 concentration for 24 h. Following treatment, cells were recovered in fresh medium and collected at the indicated time points. Genomic DNA was isolated, purified, and quantified before slot blotting. Cisplatin–DNA adducts were detected using a monoclonal antibody against cisplatin-modified DNA, and SYBR Gold staining was included as a loading control. (**B**,**E**), Quantification of cisplatin–DNA adduct accumulation in control and HMGB2-depleted cells. Band intensities were normalized first to SYBR Gold loading control and then to the 0-h siNT group for A2780 and CP70, respectively. (**C**,**F**), Analysis of cisplatin–DNA adduct repair kinetics in A2780 and CP70 cells. The percentage of remaining DNA adducts in each group was determined by normalizing band intensities to SYBR Gold and then to the corresponding 0-h time point. Data are presented as mean ± SEM from at least three independent experiments. Statistical significance was identified by ordinary two-way ANOVA with Sidak’s multiple comparisons test (** *p* < 0.01; *** *p* < 0.001; **** *p* < 0.0001; ns, not significant).

Although HMGB2 depletion enhanced cisplatin sensitivity and apoptosis in ovarian cancer cells, only limited changes in DDR signaling and DNA repair protein levels were observed. Thus, we next assessed whether HMGB2 depletion affects the formation and removal of cisplatin-induced DNA lesions. Cisplatin primarily exerts its cytotoxic effects through the formation of DNA adducts, including intrastrand lesions and ICLs, which distort DNA structure and require coordinated processing and repair for cell survival [5]. Therefore, we examined the impact of HMGB2 depletion on cisplatin–DNA adduct accumulation and repair kinetics using an immuno-slot blot assay with an antibody specific for cisplatin-modified DNA. A2780 and CP70 cells in the siNT- and siHMGB2-treated groups were treated with cisplatin at half of the IC50 concentration for 24 h and allowed to recover in drug-free medium prior to genomic DNA isolation at 0, 24, and 96 h post-treatment. Representative slot blot images are shown in Figure 4A and Figure 4D for A2780 and CP70 cells, respectively. In A2780 cells, HMGB2 depletion resulted in a significant increase in cisplatin–DNA adduct levels (210%) compared to siNT-treated cells at 0 h (Figure 4B). However, the differences between groups diminished during the recovery period at 24 or 96 h. In CP70 cells, HMGB2-deleted cells exhibited significantly higher cisplatin–DNA adduct formation at the early time points, including approximately 140% at 0 h and ~180% at 24 h relative to siNT controls (Figure 4E). These differences were no longer significant at 96 h. To determine whether HMGB2 depletion affects lesion removal kinetics, adduct levels detected at each recovery time point were normalized to the corresponding 0 h value for each treatment group (Figure 4C,F). While A2780 cells showed similar adduct removal kinetics between siNT and siHMGB2 groups, HMGB2-depleted CP70 cells exhibited significantly higher levels of cisplatin–DNA adducts remaining at 24 h post-treatment, indicating the delay in lesion removal. No significant differences were observed at the later recovery time point. These results demonstrate that HMGB2 depletion promotes early accumulation of cisplatin–DNA adducts and delays their resolution in chemoresistant ovarian cancer cells. Together with the apoptosis and clonogenic survival data, these findings support a role for HMGB2 in facilitating the efficient processing of cisplatin-induced DNA damage following treatment.

### 3.5. Depletion of HMGB2 Impairs the Processing of Cisplatin-Induced ICLs in Cisplatin-Resistant Human Ovarian Cancer Cells

Because HMGB2 depletion increased cisplatin sensitivity and promoted persistence of cisplatin–DNA adducts, we next examined whether HMGB2 contributes to the processing of cisplatin-induced ICLs, which are highly cytotoxic DNA lesions associated with cisplatin treatment. A modified alkaline comet assay was performed to evaluate ICL formation and processing following cisplatin exposure.

**Figure 5:**
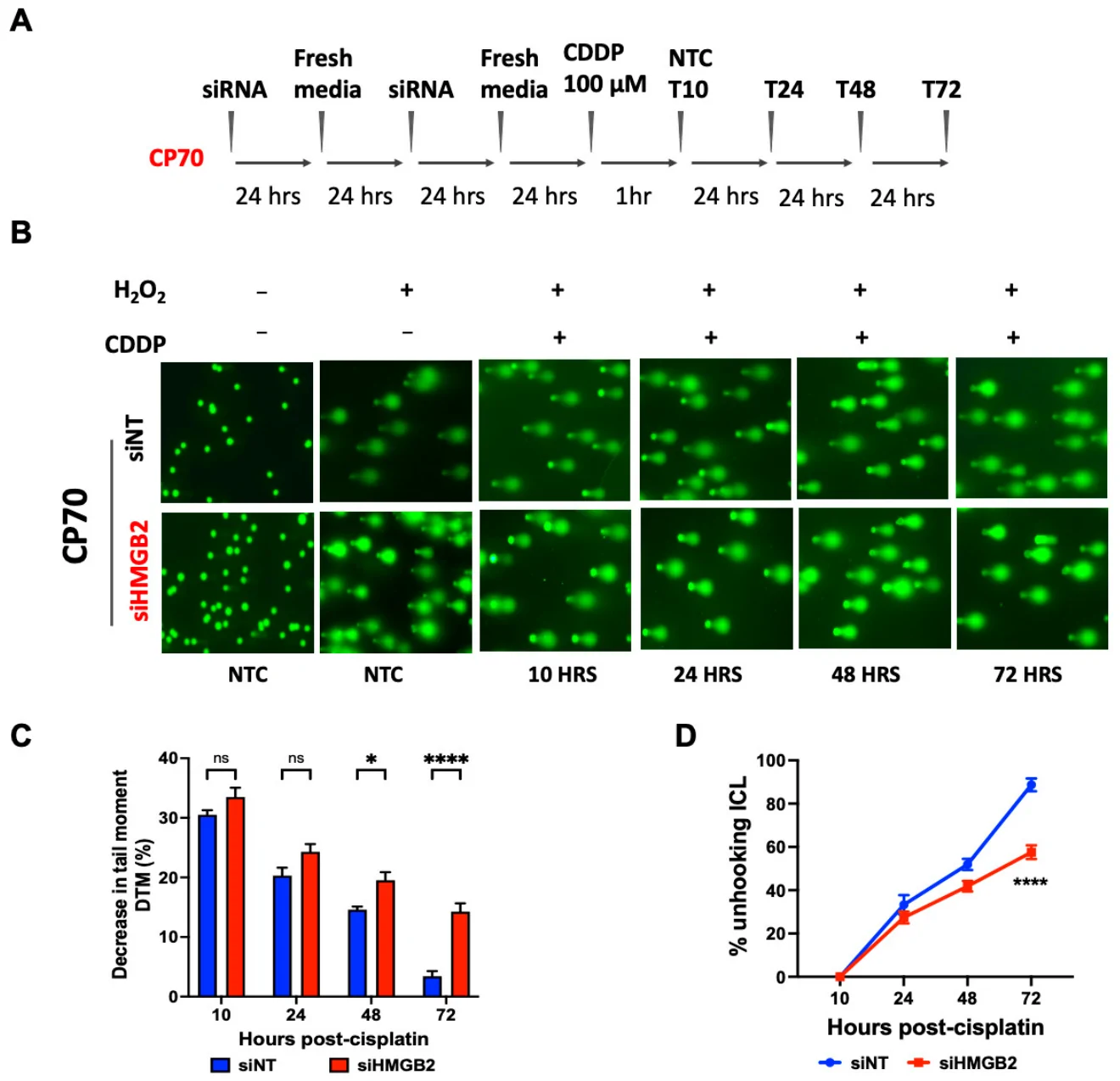
Impact of HMGB2 depletion on the processing of cisplatin-induced ICLs in human ovarian cancer cells. (**A**), Experimental outline of the modified alkaline comet assay conducted in cisplatin-resistant CP70 cells. (**B**), Representative comet images in control (siNT) and HMGB2-depleted CP70 cells following cisplatin treatment, visualized using CometScore 2.0 software. (**C**), Quantification of the percentage decrease in tail moment (%DTM) at the indicated time points post-cisplatin treatment. HMGB2-depleted cells exhibited a significantly elevated level of cisplatin-induced ICLs compared to the control cells at both 48- and 72-h time points. (**D**), The kinetics of ICL unhooking over time in siNT and siHMGB2 cells, relative to the 10-h peak value. HMGB2 depletion significantly delayed ICL processing at 72 h post-treatment. Data are shown as mean ± SEM (n ≥ 3). Statistical analysis was performed by ordinary two-way ANOVA with Sidak correction (* *p* < 0.05; **** *p* < 0.0001; ns = not significant).

In this assay, control and HMGB2-depleted cells were exposed to cisplatin, followed by recovery in drug-free medium and sample collection at the indicated time points (Figure 5A). To facilitate ICL detection, cells were treated with H_2_O_2_ immediately before the lysis step to induce random DNA strand breaks. The presence of ICLs inhibits DNA migration during electrophoresis, resulting in decreased tail moment (DTM) values relative to non-treated controls. Therefore, increased DTM values correlate with higher levels of cisplatin ICLs, whereas restoration of DNA migration over time reflects the processing of cisplatin ICLs following unhooking rather than complete ICL repair. ICL unhooking is the initial processing step of ICL repair in which the covalent linkage between the two DNA strands is released, allowing downstream repair pathways to restore DNA integrity. Representative comet images for CP70 cells are shown in Figure 5B. Data analysis of DTM demonstrated similar initial ICL induction levels between groups at 10 and 24 h post cisplatin treatment (Figure 5C). However, the DTM values in HMGB2-depleted CP70 cells were significantly higher at 48 and 72 h post-treatment compared to siNT controls, indicating higher ICL levels. To further examine repair efficiency, the percentage of ICL unhooking was calculated by normalizing the DTM value at each time point to that observed at the 10-h time point (Figure 5D). siHMGB2-depleted CP70 cells exhibited lower ICL unhooking percentages throughout the recovery period, and this difference was significant at 72 h compared with siNT cells, indicating the delay in ICL processing. Similarly, in A2780 cells, HMGB2 depletion resulted in significantly elevated ICL levels at 24 and 48 h post-treatment, along with reduced ICL unhooking at 48 h following cisplatin exposure (Supplementary Figure S2). Collectively, these findings suggest that HMGB2 depletion impairs the processing of cisplatin ICLs, which may contribute to the enhanced cisplatin sensitivity that was observed in cisplatin-resistant ovarian cancer cells.

## 4. Discussion

Cisplatin resistance remains a challenge in the treatment of ovarian cancer treatment and can contribute to tumor recurrence and poor patient outcomes. The cytotoxic effects of cisplatin occur primarily via the formation of DNA lesions, including DNA intrastrand adducts and ICLs, which disrupt DNA replication and transcription and activate multiple DDR and DNA damage repair pathways. Therefore, the ability of cancer cells to process and repair cisplatin-induced DNA damage plays an important role in determining sensitivity to cisplatin.

HMGB2 is a member of the chromatin-associated high-mobility group box (HMGB) protein family, which also includes HMGB1 and HMGB3. HMGB proteins preferentially bind distorted DNA structures, including cisplatin-modified DNA. Previous studies demonstrated that HMGB2 depletion reduced cell proliferation and increased cisplatin-induced apoptosis in hepatocellular carcinoma cells [20], while HMGB2 silencing enhanced sensitivity to cisplatin treatment in head and neck squamous cell carcinoma cells [27]. In glioma, HMGB2 expression was associated with poor prognosis, radiation resistance, and increased DNA damage repair activity [19,21]. These findings suggest that HMGB2 may be involved in cellular responses to genotoxic treatment and may modulate sensitivity to DNA-damaging therapies.

In our previous studies, depletion of HMGB1 or HMGB3 enhanced cisplatin sensitivity and affected DDR signaling following cisplatin treatment in chemoresistant human ovarian cancer cells [12,13]. HMGB1 depletion enhanced cisplatin-induced apoptosis, altered ATM/CHK2 and ATR/CHK1 signaling responses, increased cisplatin–DNA adduct formation, and impaired cisplatin–DNA adduct removal and ICL repair efficiency, particularly in cisplatin-resistant CP70 cells. HMGB1 depletion also modulated the levels of several DNA repair proteins, including ERCC1 and NBS1, suggesting a role in both DNA damage signaling and repair activities. Similarly, HMGB3 depletion increased cisplatin sensitivity in chemoresistant CP70 cells, reduced clonogenic survival, altered DDR signaling, and delayed removal of cisplatin–DNA adducts following cisplatin treatment. Together, these findings suggest that HMGB proteins contribute to cellular responses to cisplatin treatment and participate in mechanisms associated with cisplatin resistance in ovarian cancer cells.

In this study, we found that HMGB2 depletion enhanced the sub-G1 population following cisplatin treatment and increased sensitivity to cisplatin in cisplatin-resistant CP70 ovarian cancer cells (Figure 1 and Figure 2). Compared with HMGB1 and HMGB3, however, HMGB2 depletion had relatively limited effects on the phosphorylation of key DDR signaling proteins following cisplatin treatment. In A2780 cells, reductions in p-ATR, p-CHK1, and p-CHK2 were observed at selected time points, whereas only several changes in DDR signaling protein levels were detected in CP70 cells. Similarly, the levels of several DNA damage repair proteins were not changed following HMGB2 depletion in either cell line (Figure 3). These findings suggest that HMGB2 depletion has relatively modest effects on canonical DDR signaling pathways and the levels of DNA damage repair proteins following cisplatin treatment in human ovarian cancer cells.

Importantly, HMGB2 depletion significantly increased cisplatin–DNA adduct accumulation and delayed processing of these lesions, especially in chemoresistant CP70 cells following cisplatin treatment (Figure 4 and Figure 5). Slot blot and modified alkaline comet assays demonstrated that HMGB2 depletion increased the accumulation and persistence of cisplatin–DNA adducts in CP70 cells, especially cisplatin-induced ICLs. The comparable ICL levels observed at the early time points between siHMGB2 and siNT CP70 cells indicate that HMGB2 depletion does not affect the initial formation of cisplatin-induced ICLs. The differences observed at later time points suggest that HMGB2 contributes to the processing of these lesions. Because ICLs are among the most cytotoxic DNA lesions, impaired processing of these lesions likely contributed to the increased apoptosis and reduced clonogenic survival observed in HMGB2-depleted CP70 cells.

The current findings suggest that HMGB2 may contribute more directly to the processing efficiency of cisplatin-induced DNA lesions than to the regulation of DNA damage signaling pathways. Efficient repair requires not only the activation of checkpoint signaling but also appropriate chromatin accessibility, the recruitment of repair proteins, the coordination of repair complexes, and the processing of repair intermediates. In this context, HMGB2 as an architectural chromatin-associated protein, which can bend DNA, alter chromatin organization, and facilitate protein–DNA interactions [11,28,29], may influence the accessibility of the repair machinery to damaged DNA, especially ICLs. Therefore, depletion of HMGB2 may impair lesion resolution and lead to persistent DNA damage.

Interestingly, although depletion of HMGB1, HMGB2, and HMGB3 produced several overlapping effects, including enhanced cisplatin sensitivity, increased sub-G1 populations consistent with apoptosis, modulation of the expression levels of DDR signaling and repair proteins, and impaired processing of cisplatin-induced DNA lesions, the extent and pattern of these effects were not identical among the three HMGB proteins. HMGB1 depletion led to more pronounced alterations in DDR signaling and DNA repair protein levels, as well as reduced repair of cisplatin DNA lesions [12]. HMGB3 depletion also altered ATR/CHK1 DDR signaling responses and delayed cisplatin–DNA adduct removal [13]. In contrast, HMGB2 depletion showed a stronger association with persistence and impaired processing of cisplatin-induced DNA lesions, particularly ICLs, despite relatively limited effects on the levels and phosphorylation of examined DDR signaling proteins. These findings suggest that HMGB family proteins may contribute to common cellular responses to cisplatin-induced DNA damage via partially overlapping and/or non-identical mechanisms.

Collectively, these studies reveal the roles of HMGB family proteins in modulating cisplatin response and chemoresistance in human ovarian cancer cells. These findings suggest that HMGB proteins contribute to cellular tolerance to cisplatin-induced DNA damage by regulating DNA damage signaling, lesion processing, chromatin-associated repair activities, and/or survival following genotoxic stress. Importantly, these studies also highlight the potential significance of chromatin-associated mechanisms in determining cisplatin response and suggest that HMGB-associated pathways may represent therapeutic targets to improve cisplatin sensitivity in chemoresistant ovarian cancer cells.

Although the current study demonstrates that HMGB2 depletion impairs cisplatin lesion processing and enhances cisplatin sensitivity, the exact molecular mechanisms underlying these effects remain unclear. Future studies examining interactions between HMGB proteins and specific repair protein complexes, or chromatin remodeling activities following cisplatin treatment, may further clarify the mechanistic contributions of individual HMGB family members in cellular responses to cisplatin. In addition, the present study did not investigate DNA damage tolerance pathways. For example, the persistence of cisplatin-induced DNA lesions despite relatively modest changes in DNA damage response signaling and DNA repair protein levels suggests that additional mechanisms may contribute to the observed phenotype. One potential mechanism is translesion synthesis (TLS), which enables replication to proceed across persistent DNA lesions. Future studies are needed to determine whether HMGB2 depletion impairs DNA damage tolerance pathways, such as TLS, thereby contributing to the persistence of cisplatin-induced DNA lesions observed in this study.

Overall, our findings identify HMGB2 as a contributor to cisplatin-induced DNA lesion processing and cellular responses to cisplatin treatment in human ovarian cancer cells. Together with our previous studies on HMGB1 and HMGB3, the current work provides additional insight into the roles of chromatin-associated factors in modulating cellular responses to cisplatin-induced DNA damage and cisplatin chemoresistance.

## Data Availability

The data supporting the findings of this study are contained within the article and the Supplementary Materials.

## Supplementary Materials

The following supporting information can be downloaded at: https://www.mdpi.com/article/10.3390/genes17080916/s1, Figure S1: HMGB2 depletion affects DNA repair protein levels following cisplatin treatment in A2780 and CP70 cells. (A,B) Western blot analysis of γH2AX, XPF, ERCC1, and NBS1 following cisplatin exposure in siHMGB2 or siNT-treated A2780 and CP70 cells. β-actin was used as the loading control. (C,D) Quantification of normalized protein levels in siNT and siHMGB2 groups. Band intensities were first normalized to β-actin and then to the 0-hour in the siNT group. Data represent mean ± SEM from ≥3 independent replicates. Statistical analysis was conducted using two-way ANOVA with Sidak’s correction (* *p* < 0.05; ns, not significant); Table S1: List of antibodies used in Western blotting; Figure S2: HMGB2 depletion impacts the formation and repair of cisplatin-induced ICLs in A2780 cells. A, A2780 cells transfected with siNT or siHMGB2 were subjected to the modified alkaline comet assay. B, The percentage of decrease in tail moment (%DTM) was quantified across the indicated time points to evaluate ICL accumulation. C, The percentage of ICL unhooking was calculated relative to the 10-hour time point to assess repair kinetics. Data represent mean ± SEM from three independent replicates. Statistical analysis was performed using two-way ANOVA with Sidak’s multiple comparisons test (* *p* < 0.05; ** *p* < 0.01; ns, not significant).
