# Supplementary material for: HMGB2 Impacts Cisplatin-Induced DNA Adduct Processing in Chemoresistant Ovarian Cancer Cells
Source: Genes (Basel). 2026 Aug 2;17(8):916. doi: 10.3390/genes17080916 (PMC13512672; doi:10.3390/genes17080916)
Supplement: Supplementary file 1 [file genes-17-00916-s001.zip › genes-4466495-supplementary.pdf]

Supplementary Table S1: List of antibodies used in western blotting

Antibody	Manufacturer	Catalog Number	RRID	Host Species	Dilution
P-ATM	Abcam	ab81292	RRID:AB_1640207	Rabbit	1:5000
ATM	Cell Signaling Technology	2783	RRID:AB_2062659	Rabbit	1:1000
P-ATR	GeneTex	GTX128145	RRID:AB_2687562	Rabbit	1:500
ATR	Santa Cruz Biotechnology	sc-515173	RRID:AB_2893291	Mouse	1:200
P-CHK1	Cell Signaling Technology	2344	RRID:AB_331488	Rabbit	1:500
CHK1	Santa Cruz Biotechnology	sc-8408	RRID:AB_627257	Mouse	1:500
P-CHK2	Cell Signaling Technology	2661	RRID:AB_331479	Rabbit	1:500
CHK2	Cell Signaling Technology	2662	RRID:AB_2080793	Rabbit	1:500
HMGB1	Abcam	ab18256	RRID:AB_444360	Rabbit	1:5000
HMGB2	Abcam	ab67282	RRID:AB_1140885	Rabbit	1:1000
γ-H2AX	Sigma-Aldrich	07-164		Rabbit	1:500
XPF	Cell Signaling Technology	13465	RRID:AB_2798227	Rabbit	1:1000
ERCC1	BD Pharmingen	556452		Mouse	1:200
NBS1	Novus Biologicals	NB 100-143	RRID:AB_350080	Rabbit	1:1000
β-actin	enQuire BioReagents	QAB10339		Rabbit	1:5000

Supplementary Data:

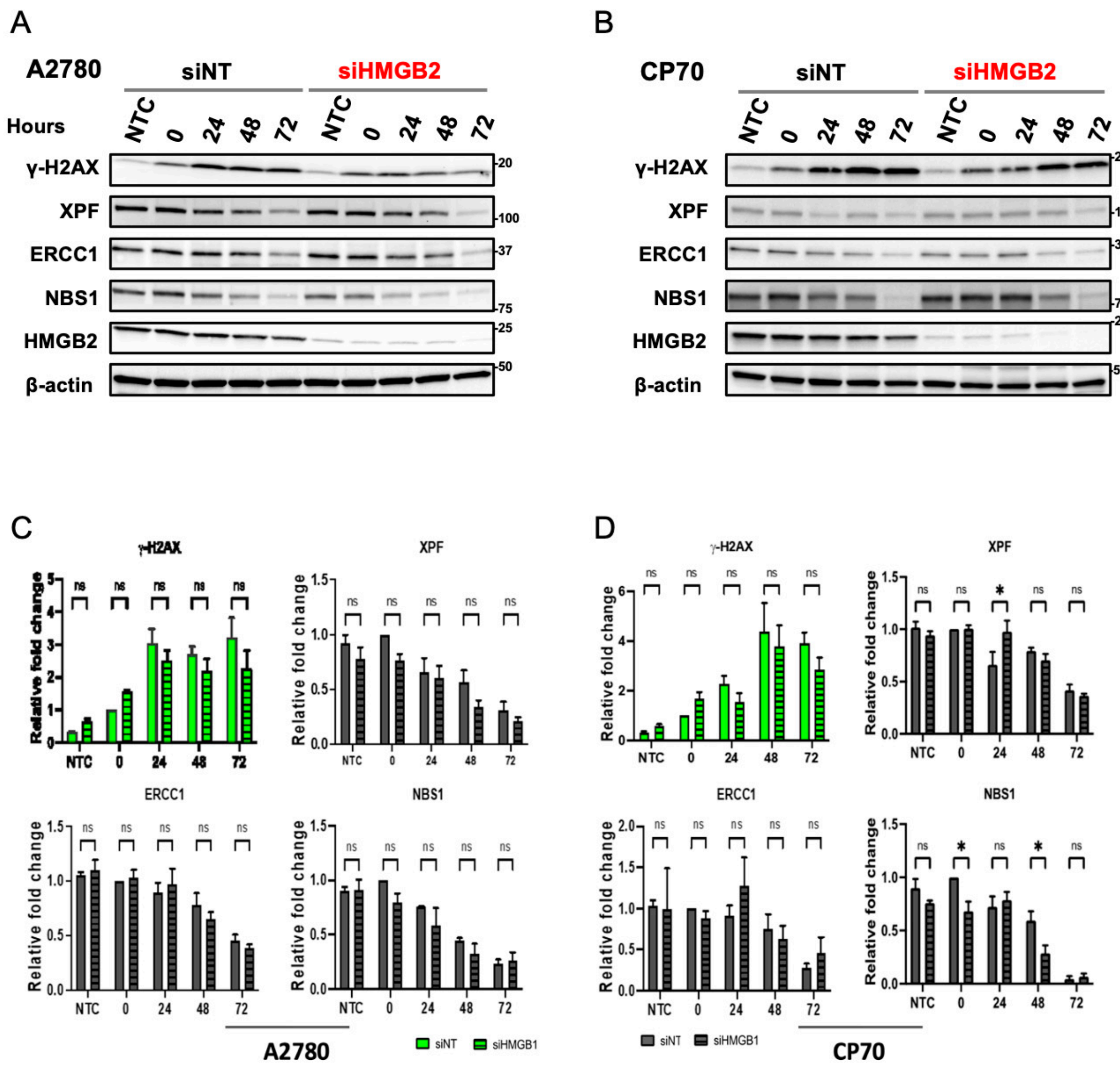

Supplementary Figure S1. HMGB2 depletion affects DNA repair protein levels following cisplatin treatment in A2780 and CP70 cells. A, **B**, Western blot analysis of γH2AX, XPF, ERCC1, and NBS1 following cisplatin exposure in siHMGB2 or siNT-treated A2780 and CP70 cells. β-actin was used as the loading control. **C**, **D**, Quantification of normalized protein levels in siNT and siHMGB2 groups. Band intensities were first normalized to β-actin and then to the 0-hour in the siNT group. Data represent mean ± SEM from ≥3 independent replicates. Statistical analysis was conducted using two-way ANOVA with Sidak’s correction ($p < 0.05$; *$p < 0.01$; **$p < 0.001$; ***$p < 0.0001$; ns, not significant).

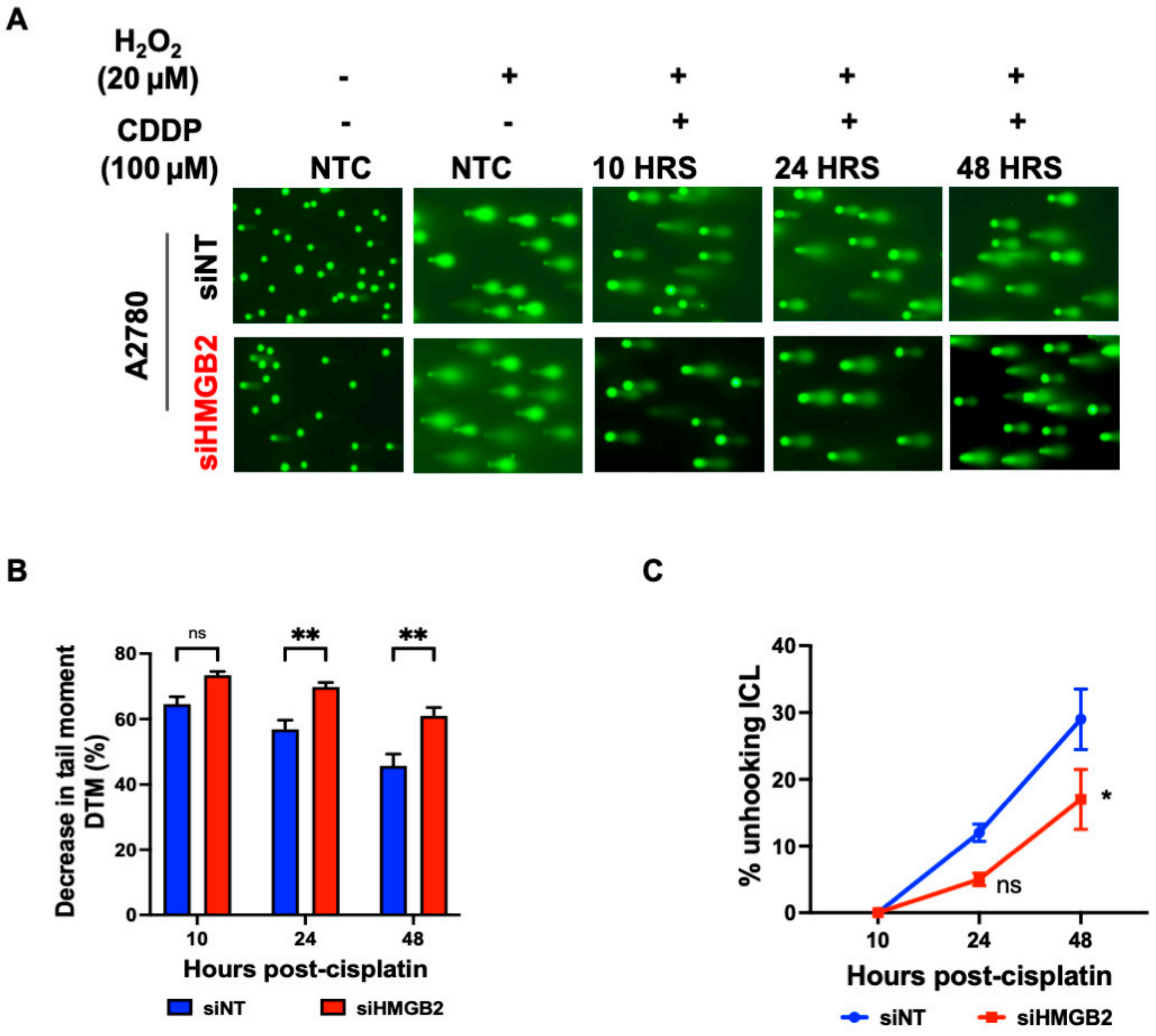

Supplementary Figure S2. HMGB2 depletion impacts the formation and repair of cisplatin-induced ICLs in A2780 cells. A, A2780 cells transfected with siNT or siHMGB2 were subjected to the modified alkaline comet assay. **B,** The percentage of decrease in tail moment (%DTM) was quantified across the indicated time points to evaluate ICL accumulation. **C,** The percentage of ICL unhooking was calculated relative to the 10-hour time point to assess repair kinetics. Data represent mean ± SEM from three independent replicates. Statistical analysis was performed using two-way ANOVA with Sidak's multiple comparisons test ($p < 0.05$; *$p < 0.01$; **$p < 0.001$; ***$p < 0.0001$; ns, not significant).
